# Transcriptome dynamics of *Gossypium purpurascens* in response to abiotic stresses by Iso-seq and RNA-seq data

**DOI:** 10.1038/s41597-024-03334-9

**Published:** 2024-05-09

**Authors:** Abdul Rehman, Chunyan Tian, Shoupu He, Hongge Li, Shuai Lu, Xiongming Du, Zhen Peng

**Affiliations:** 1 Zhengzhou Research Base, State Key Laboratory of Cotton Bio-breeding and Integrated Utilization, School of Agricultural Sciences, Zhengzhou University, Zhengzhou, 450001 China; 2 State Key Laboratory of Cotton Bio-breeding and Integrated Utilization, Institute of Cotton Research, Chinese Academy of Agricultural Sciences (ICR, CAAS), Anyang, Henan 455000 China; 3 National Supercomputing Center in Zhengzhou, Zhengzhou University, Zhengzhou, 450001 China; 4 National Nanfan Research Institute (Sanya), Chinese Academy of Agricultural Sciences, Sanya, Hainan 572024 China

**Keywords:** Transcriptomics, Agricultural genetics

## Abstract

*Gossypium purpurascens* is a member of the Malvaceae family, holds immense economic significance as a fiber crop worldwide. Abiotic stresses harm cotton crops, reduce yields, and cause economic losses. Generating high-quality reference genomes and large-scale transcriptomic datasets across diverse conditions can offer valuable insights into identifying preferred agronomic traits for crop breeding. The present research used leaf tissues to conduct PacBio Iso-seq and RNA-seq analysis. We carried out an in-depth analysis of DEGs using both correlations with cluster analysis and principal component analysis. Additionally, the study also involved the identification of both lncRNAs and CDS. We have prepared RNA-seq libraries from 75 RNA samples to study the effects of drought, salinity, alkali, and saline-alkali stress, as well as control conditions. A total of 454.06 Gigabytes of transcriptome data were effectively validated through the identification of differentially expressed genes and KEGG and GO analysis. Overwhelmingly, gene expression profiles and full-length transcripts from cotton tissues will aid in understanding the genetic mechanism of abiotic stress tolerance in *G. purpurascens*.

## Background & Summary

Abiotic stresses, such as water deficit, high pH, and salt accumulation, can significantly impact soil health, leading to a decline in crop quality and yield. This can pose a severe threat to food security, highlighting the need for sustainable agricultural practices to mitigate the effects of these stresses. In response to abiotic stresses, plants undergo a multifaceted and intricate set of reactions that involve a range of molecular, physiological, and cellular changes in various plant tissues^1^. Various breeding methods have been employed to comprehend how plants react to abiotic stresses, ranging from conventional approaches to advanced -omics methods like next-generation sequencing (NGS).

Single-molecule long-read sequencing, also known as PacBio Iso-seq or third-generation sequencing, is a powerful technology that can be used to accurately identify full-length RNA transcripts^2^. This approach has numerous technical advantages over traditional sequencing methods and is particularly useful for analyzing complex transcriptomes. By providing long reads that span entire transcripts, PacBio Iso-seq enables the identification of novel isoforms, splice variants, and other structural features that are often missed by short-read sequencing. Moreover, this technology can be combined with other sequencing methods to generate comprehensive and accurate transcriptome annotations^2^. The PacBio sequencing system can currently sequencing transcripts up to 30 kb in their full length. However, the sequencing depth is low (Our dataset comprises approximately 0.4 million full-length non-chimeric reads per sample) and there is a high error rate in base calling, which is approximately 15%^3,4^. The Illumina paired-end RNA-seq technique, a type of second-generation sequencing, helps fragment RNAs into reads with significantly higher depth and accuracy. Our data showed that each sample generated around 20 million paired-end reads^5,6^. By leveraging the strengths of both long- and short-read sequencing, we can achieve more significant insights into isoform diversity while obtaining precise quantitative measurements. NGS has paved the way for extensive exploration of the transcriptome, enabling us to delve deeper into the molecular mechanisms underlying the adaptive responses of various plant species to their surroundings. At present, the analysis of transcriptome data in plants is being carried out in different organisms, and with varying conditions, encompassing the evaluation of responses to abiotic stresses. The majority of the transcriptome analyses conducted on abiotic stress responses have been carried out in model plant systems. Hence, we aim to investigate the transcriptional regulatory mechanisms of semi wild cotton species towards diverse stress conditions.

Upland cotton (*Gossypium hirsutum* L.) is the most widely grown and utilized source of renewable textile fiber, contributing to over 90% of the world’s fiber production. It is an allotetraploid species that originated from a single hybridization event approximately 1–1.5 million years ago. Cotton has been cultivated for over 7,000 years and is a crucial crop for the textile industry^7,8^. Continuously narrowing down the genetic diversity has resulted in a decline in the quality of fibers. To overcome this issue, exploring and utilizing the genetic variation present in landraces and wild genotypes is crucial as they contain distinct elite alleles that can improve the overall quality of fibers^9–11^. Using wild progenitors and landraces is a promising approach for generating desirable genetic variations in contemporary cultivars. Such genetic materials possess unique traits that can be harnessed through selective breeding or advanced genetic techniques to improve the productivity, resilience, and adaptability of the existing cultivars. This strategy has proven successful in many crop species and is gaining popularity among plant breeders and geneticists as an effective means of crop improvement^12^. *Gossypium hirsutum* L. *purpurascens*, a tetraploid cotton landrace, is a perennial plant extensively cultivated in several regions during the 17th century, including China, Brazil, India, Africa, Congo, and Egypt^13,14^. During the classification of *Gossypium purpurascens*, experts had divergent opinions. Harland and Watt have classified it as a distinct species of the *Gossypium* genus, while two other researchers, Hutchinson and Stephens, believe it is a *Gossypium hirsutum* landrace. The identification of *G. purpurascens* has been a debate among botanists and cotton breeders due to its morphological and genetic characteristics that show both similarities and differences with other cotton species^13,15–17^. Following its discovery, genetic analysis revealed that *Gossypium purpurascens* did not fall within the seven known geographical landraces of *G. hirsutum* as classified by the scientific community. The provenance and chronology of these indigenous cultivars remain unreported. Therefore, we believe that *G. purpurascens* is an overlooked variety that harbors significant natural diversity. *G. purpurascens* is a plant species that exhibits photoperiodic sensitivity. In contrast, due to unfavorable climatic conditions prevailing in North and Central China, the reproductive phase of the plant cannot occur naturally. Due to this limitation, the species is predominantly found in the province of Hainan, China. The aforementioned species was successfully introduced in the southern Chinese provinces of Guangxi, Fujian and Guangdong, known for their favorable environmental conditions. The *G. purpurascens* species found in various islands of South China, including Sansha and Naozhou Island in Hainan and Guangdong Province, respectively, is morphologically distinct and geographically isolated. It has been identified as a wild-type *G. hirsutum*^18^.

According to the genomic analysis of *G. purpurascens*, it has been discovered that this primitive species possesses a remarkable resistance towards saline-alkali stress^19^. This reveals a fascinating insight into the adaptive capabilities of this species, and sheds light on its ability to survive and thrive in harsh and challenging environments. Peng *et al*. conducted a study on 45 genotypes of *G. purpurascens* and found that most of them could resist salt^20^. This corroborates with the findings mentioned earlier. Hence, this study represents the first known instance of long-read sequencing and short read sequencing conducted in parallel to investigate the effects of salinity, drought, alkali, and saline-alkali stress on *G. purpurascens*. Present transcriptomic data is a valuable resource for researchers seeking to deepen their understanding of the molecular mechanisms behind drought, salt, and saline-alkali tolerance in *G. purpurascens*.

## Methods

### Plant materials and treatment

**Fig. 1:**
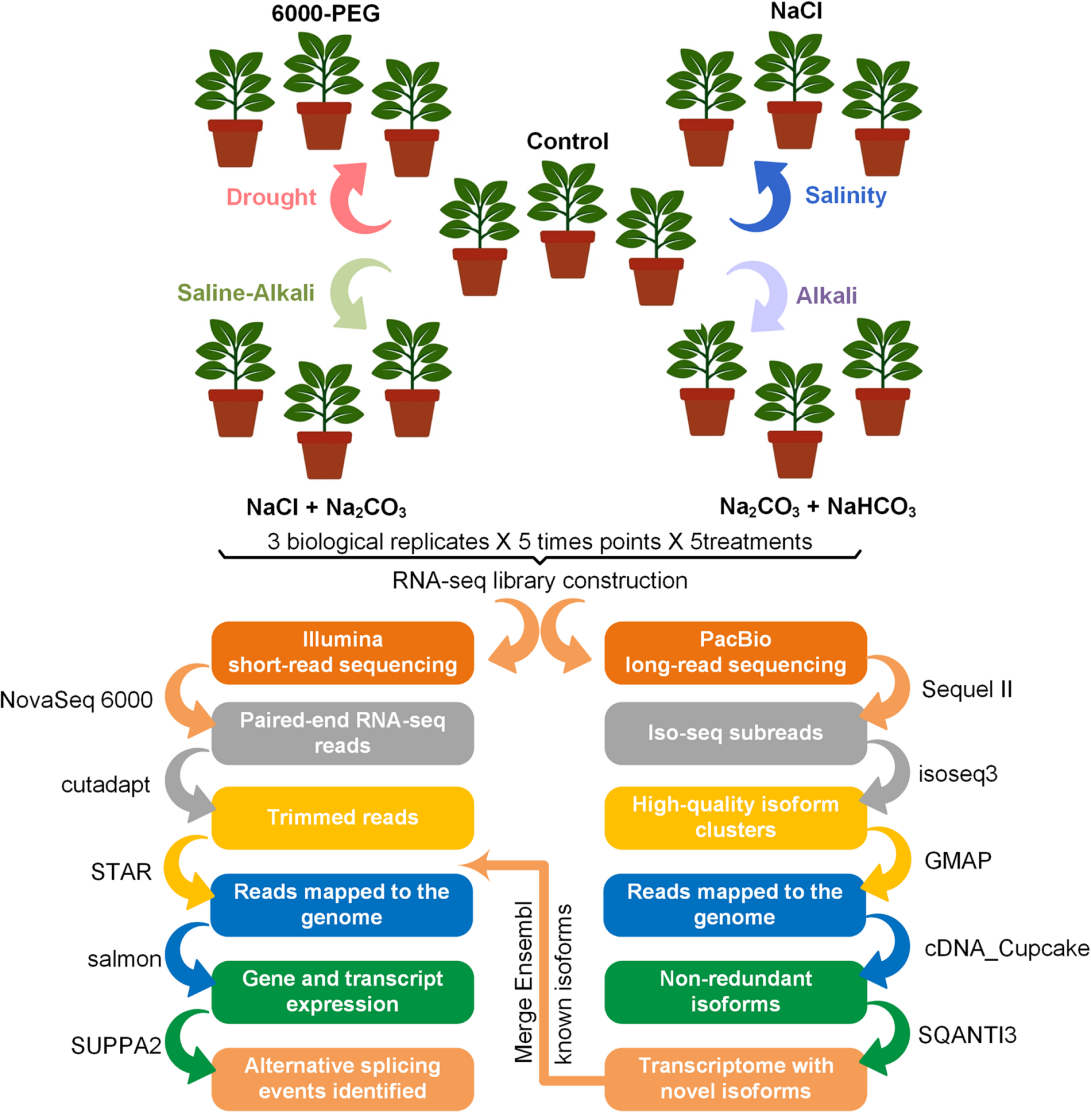
Summary of experimental design, sample collection, and data analysis workflow in PacBio long read and Illumina short read sequencing of *Gossypium purpurascens* in drought, salinity, alkali, and saline-alkali stress. (Preferably placed before Experimental design overview).

Salt and saline-alkali resistant genotype of *G. purpurascens* (K411: Zhanjiang Naozhou Liuluo-2) were used for Iso-seq and RNA seq^19,20^. Furthermore, it should be noted that this specific genotype has undergone prior investigation, and was determined to possess resistance to high levels of salt^20^. After two weeks of germination, the seedlings obtained from K411 were transferred to a 32-plug tray with a diameter of 6 cm and a height of 6.5 cm. It was ensured that each of the seedlings was carefully uprooted and replanted into the tray to promote their healthy growth. The growth room was maintained at a temperature of 24 ± 1 °C with a photoperiod of 16 hours light and 8 hours dark. At the stage when the plants had four true leaves, a drought stress experiment was conducted by applying a 15% solution of 6000-poly ethyl glycol (PEG-6000) in a quantity of 100 ml. A 0.4% (0.4 g NaCl:100 g sand) NaCl solution was administered to induce salinity stress in plants. For alkali stress, cotton plants were treated with a solution containing 0.42 g NaHCO_3_ and 0.53 g Na_2_CO_3_ per 100 g of sand, with a total volume of 100 mL. The experiment also involved subjecting plants to saline-alkali stress by treating them with NaCl and NaHCO_3_. The solution was prepared by mixing 0.585 g of NaCl and 0.53 g of Na_2_CO_3_ in 100 mL of water. The plants were exposed to this solution simultaneously to induce the desired stress conditions. To perform transcriptome profiling, three to four leaves were harvested from randomly selected four plants per replicate at various time points i.e., 0.5, 3, 12, 24, and 48 hours after treatment (Fig. 1). We collected three biological replicates for each experimental condition at every time point for accurate and reliable data collection. Following the plucking of the leaves, they were rapidly frozen with liquid nitrogen and preserved at an ultra-low temperature of −80 °C until the extraction of RNA.

### Experimental design overview

In order to obtain reliable and accurate results, we collected 75 samples at the fourth leaf stage from four different cotton plants for each biological replicate. The leaves were collected following a specific treatment and mock control at different time intervals of 0.5, 3, 12, 24, and 48 hours. After that, libraries for PacBio sequencing and RNA sequencing were prepared and subjected to sequencing. Transcriptome data was subjected to a quality assessment and mapped to the genome of *G. purpurascens* (HPF17)^19^. Figure 1 illustrates the workflow of the pipeline used for the treatment of abiotic stress and the analysis of transcriptome data.

### Extract RNA, construct library, and sequenceing

Following the manufacturer’s instructions, 100 mg of cotton leaf samples were subjected to RNA extraction using Trizol reagent (Ambion, USA). The extracted RNA was quantified and qualified for downstream applications. We used a NanoDrop 2000 spectrophotometer (Termo Scientifc, USA) to quantify the RNA spectrophotometrically for RNA quality control. Additionally, we verified RNA integrity using agarose gel electrophoresis. Five micrograms of each RNA sample were used to generate a strand-specific library containing inserts of approximately 150–200 bp. A comprehensive transcriptome analysis was carried out on a total of 75 cDNA libraries obtained from five distinct treatment groups. The study involved a detailed examination of the expressed genes and their corresponding transcriptional activity in each library. The treatments included four abiotic stresses and a mock control. Each treatment was analyzed separately to identify any significant changes in gene expression (Table 1). The NovaSeq. 6000 platform (Illumina, USA) performed RNA sequencing with paired-end sequencing of 150 nucleotides.

**Table 1:** Statistical summary of the data in *Gossypium purpurascens*.

Description	Total Read numbers/Average length (bp)
CCS Number	501,978
Mean Read Length of CCS	1,552
Read Bases of CCS	779,075,023
FLNC Number	1,523
Mean Read Length of FLNC	149,108
Full-length non-chimeric (FLNC) reads	421,121
FLNC sequences	149,108
High-quality consensus sequence	149,062
Final transcripts	89,837
Fusion transcripts	612
Total transcripts	89,837
Gene loci	43,548
Novel gene loci	10,477
Novel transcripts	65,666
SSRs	38,640
ORFs	38,406
lncRNAs	1,522

(Preferably placed before Constructing and sequencing of PacBio libraries).

### Constructing and sequencing of PacBio libraries

For the preparation of PacBio sequencing library, we combined RNA extracted from 75 leaf tissue samples (RNA samples corresponding to 75 RNA-seq libraries) and performed reverse transcription to synthesize cDNA. To achieve this, a PCR cDNA Synthesis Kit of SMARTer® was utilized, which enabled the generation of high-quality cDNA libraries for downstream sequencing applications. The BluePippin size selection system, manufactured by Sage Science in the USA, was employed to obtain PCR-amplified products. The system allowed for the isolation of fragments with a length ranging from 0.5 to 6 kb, which were subsequently used for library construction. The SMRTbell libraries used in advanced genomic research were precisely prepared using the high-quality DNA Template Prep Kit 2.0 of Pacific Biosciences. The sequencing process was carried out using polymerase 2.0 and the state-of-the-art PacBio Sequel platform, ensuring accurate and reliable results.

### Pacific Biosciences Long Read processing

The subreads in their raw form were subject to analysis using the Iso-Seq. 3 pipeline, which can be accessed at https://github.com/PacificBiosciences/IsoSeq. The initial steps of the pipeline involved the creation of circular consensus sequences (CCS) subreads, categorization of full length (FL) reads, and grouping of full-length non-chimeric (FLNC) reads. Using the CCS v6.2.0 software, we generated polished CCS subreads from the subreads bam files. These subreads had a minimum quality of 0.9, as specified by the parameter –min-rq 0.9. To generate CCS using a zero-mode waveguide (ZMW), the default value of FL subreads (n = 3) was utilized. The FL subreads refer to full-length subreads obtained from the sequencing of a template DNA molecule and are necessary to generate high-quality CCS. The FL transcripts were identified based on the presence of poly(A) tails and the use of specific 5′ and 3′ cDNA primers during sequencing. The process of primer removal was carried out using the Lima v2.1.0 tool while the isoseq. 3 performed the elimination of poly(A) tails refining technique. The process of generating superior-quality FL consensus sequences involved the application of the clustering algorithm ICE. FL consensus sequences of superior quality were classified using a stringent criterion of post-correction accuracy surpassing 99%.

### Move redundant

The consensus sequences obtained from FL were aligned to the reference genome of *G. purpurascens* using minimap by configuring it with parameters such as splice, no-C5, and uf for optimal alignment results^19^. The initially mapped reads have undergone a further collapsing process using cDNA-Cupckae, with a minimum coverage of 85% and a minimum identity of 90%.

### Structure analysis

The process of validating transcripts against known reference transcript annotations was carried out using the MatchAnnot Python library. The AS Talavista tool was able to identify multiple events, such as ES, MEE, IR, AD, and AA, related to alternative splicing. The identification of Simple Sequence Repeats (SSR) in the transcriptome was carried out using MISA, which is a software tool designed for SSR detection in DNA, RNA, and protein sequences (http://pgrc.ipk gatersleben.de/misa/misa.html). Alternative Polyadenylation (APA) analysis was also performed using TAPIS (https://bitbucket.org/comp_bio/tapis/overview). TAPIS is a tool that identifies and quantifies transcript isoforms containing alternative polyadenylation sites.

### Function annotation of unigenes

To annotate the functional unigenes of *G. purpurascens*, we searched 8 databases, which included NR^21^, KOG^22^, Swiss-Prot^23^, Pfam^24^, GO^25^, KEGG^26^, egg NOG^27^, and COG^28^. The Diamond BLASTX techniques were utilized to analyze the data with an E-value threshold of less than 1 × 10^−10^. The analysis was conducted on COG, NR, KEGG, and Swiss-Prot annotations. The Pfam database was used to perform the Hmmscan procedure, followed by the WEGO method to categorize GO functions.

### Long non-coding RNAs (lncRNAs) and predictions of open reading frames (ORFs)

To identify the ORFs in the transcripts of *G. purpurascens*, we utilized the TransDecoder v2.0.1 package (https://transdecoder.github.io/). Full-length transcripts are identified as transcripts containing both the untranslated regions (UTRs) and ORFs at their 5′ and 3′ ends. To predict the presence of lncRNAs in the transcriptome, we utilized four software programs, namely PLEK^29^, CPC^30^, CNCI^31^, and CPAT^32^.

### Illumina library construction and sequencing

In order to extract mRNAs from leaf tissues of *G. purpurascens*, a Dynabeads oligo (dT) kit from Invitrogen was utilized per the manufacturer’s instructions. This process involved the use of total RNA. During the cDNA synthesis process, Superscript II reverse transcriptase from Invitrogen was used along with random hexamer primers. The process involved the synthesis of both first and second-strand cDNA to obtain a complimentary copy of the RNA sequence. The process involved the fragmentation of double-stranded cDNA through nebulization, followed by the creation of RNA-seq libraries. The Illumina Hiseq X Ten program was employed to sequence the cDNA libraries, generating paired-end reads of 150 bp. For the RNA-seq experiments, three biological replicates were performed to ensure the accuracy and reliability of the results. The data analysis techniques of principal component analysis (PCA) and sample hierarchical clustering were applied using the “prcomp” and “cor” functions from the stats package of R. These techniques allowed for the identification of patterns and relationships within the data, enabling deeper insights and understanding.

### Illumina RNA-Seq data processing

To ensure high-quality results, we implemented a rigorous filtering and trimming process for the raw RNA sequences. We utilized cutadapt16 and the NGS QC Toolkit to remove low-quality bases and adapter sequences, which can negatively impact downstream analysis. This step was essential for generating accurate and reliable data for our research. The reads obtained from filtering were processed further and trimmed to remove any unwanted data. The resulting trimmed reads were then subjected to quality assessment using a tool called FastQC (https://www.bioinformatics.babraham.ac.uk/projects/fastqc/). The quality results obtained were merged using multiQC with default parameters to obtain an overall quality report for the processed reads. Our objective was to obtain high-quality clean reads, and we achieved this by eliminating reads that contained low-quality reads, adapter sequences, and poly-N sequences. The clean data was subjected to calculations for GC-content, Q20, Q30, and sequence duplication level simultaneously. The downstream analyses were performed using data that had undergone rigorous quality control measures, ensuring high accuracy and reliability. The reads of superior quality were aligned with the reference genome sequence to aid in conducting thorough investigations. This alignment process enables researchers to identify the location and characteristics of specific genetic variations and mutations, thus allowing for a better understanding of the genomic landscape^19^. The sequencing data was filtered to consider only the reads with a perfect match or one mismatch, and then obtained sequences were subsequently matched to the reference genome *G. purpurascens* utilizing the Hisat2 software tools.

### Quantification of gene expression levels and differential expression analysis

Gene expression levels are commonly quantified using FPKM, which normalizes the number of fragments mapped to a transcript by length and the total number of fragments mapped. We performed differential expression analysis using the edgeR package with three specific functions - estimateDisp, glmQLFTest, and glmQLFit^33^. These functions were essential in determining the differences in gene expression between our samples. All the individual factors, including time points and treatments, were merged into a single factor to develop a model formula for the experiment. This allowed for greater ease in analyzing and interpreting the experiment results. We have successfully identified genes that exhibit differential expression between the treatment and the mock groups across all time points under consideration. The identification of genes with significant DEGs between two distinct groups was carried out using a set of criteria that included an adjusted p-value of less than 0.05 and a fold change (FC) of greater than 2. The sets of DEGs were accumulated for every time point in the treatments, and the DE gene sets were combined across the different time points. To visualize the top forty-five DEGs associated with each stress, a heatmap was created using the pheatmap package^34^. The heatmap provides a graphical representation of the expression levels of these genes, allowing for easy identification of patterns and relationships between the genes and the stress conditions. To determine the functional significance of DEGs, GOseq R packages were utilized to perform Gene Ontology (GO) enrichment analysis (adjusted *p-value < *0.05). Additionally, we used KOBAS software to perform enrichment analysis of DEGs in the pathways of KEGG^35^. Overall, these analyses gave us insights into the biological processes and DEGs associated pathways.

## Data Records

The unprocessed sequence data presented in this paper has been successfully deposited in the Genome Sequence Archive^36^. The archive is maintained by the National Genomics Data Center^37^, a part of the China National Center for Bioinformation and Beijing Institute of Genomics, Chinese Academy of Sciences. The GSA accession number that corresponds to this dataset can be found publicly under CRA014488^38^. Full length transcriptome sequences file of *G. purpurascens*^39^, the full length transcriptome assembly annotation file^40^, GO ontology and KEGG pathway analysis for the annotated sequences^41^, splice data^42^, alternative polyadenylation sites^43^, SSRs^44^, predicted novel long non-coding RNA and novel isoform transcriptome sequences^45^, novel coding isoforms annotation^46^, and the data includes information on the expression levels of genes and their isoforms, as well as the classification and sequences of transcription factors^47^ can be retrieved from the database of figshare.

## Technical Validation

### Pacbio Iso-seq data processing

The PacBio ISO-seq sequencing provided comprehensive results with a total of 779,075,023 subreads that were successfully produced and analyzed (Table 1). After undergoing a self-correction, a total of 501,978 CCSs were successfully generated. These CCSs represent a highly accurate and reliable dataset that can be used for further analysis and research. The total count of FLNC reads analyzed by CCS was 421,121, with 1,552 an average read length. To refine the transcriptome assembly and improve the accuracy of the transcripts, the ICE method was employed. This involved utilizing FLNC reads to conduct a thorough analysis and obtain highly refined and polished transcripts. The high-quality consensus isoforms of RNA transcripts were obtained by polishing the transcripts with RNA-seq data through LoRDEC^48^ and eliminating redundancy. The final output consisted of 149,062 consensus isoforms with an average length of 1,523 and an accuracy ratio exceeding 99% (Table 1). The transcript data sets utilized for further analysis displayed a remarkable level of integrity, as evidenced by their non-redundancy and full-length nature.

### Quality control

FastQC and multiQC18 software were used to analyze the mean quality score of the RNA-seq data of each sequence, the GC content, and the distribution of read length. These metrics provide a comprehensive assessment of the quality and integrity of the RNA-seq data (Supplementary Table S1). It is worth mentioning that in the study conducted, all of the analyzed samples showed a significantly high level of accuracy in their sequencing, with over 90% of the sequences achieving a quality score of Q30 or above. The high level of precision achieved in the data indicates that the margin of error is minimal, which makes it extremely dependable for subsequent analysis and interpretation. Notably, a significant proportion (88%) of the samples displayed sequencing quality Q30 values exceeding 93%. The Q30 value indicates that the DNA sequencing process was exceedingly precise and dependable, with a remarkably low error rate of only 0.1%. The significance of this data cannot be overstated as it serves as a critical determinant of the accuracy and dependability of the genetic data acquired from the samples. The normal distribution of GC content across all samples suggests that there was no sequence contamination during the sequencing process. This indicates the high-quality raw reads, and the overall statistics support this conclusion. The preprocessed reads were aligned with a high mapping rate, with an average of 96.60% for mapped reads and 90.32% for uniquely mapped reads.

### Transcriptome data analysis

**Fig. 2:**
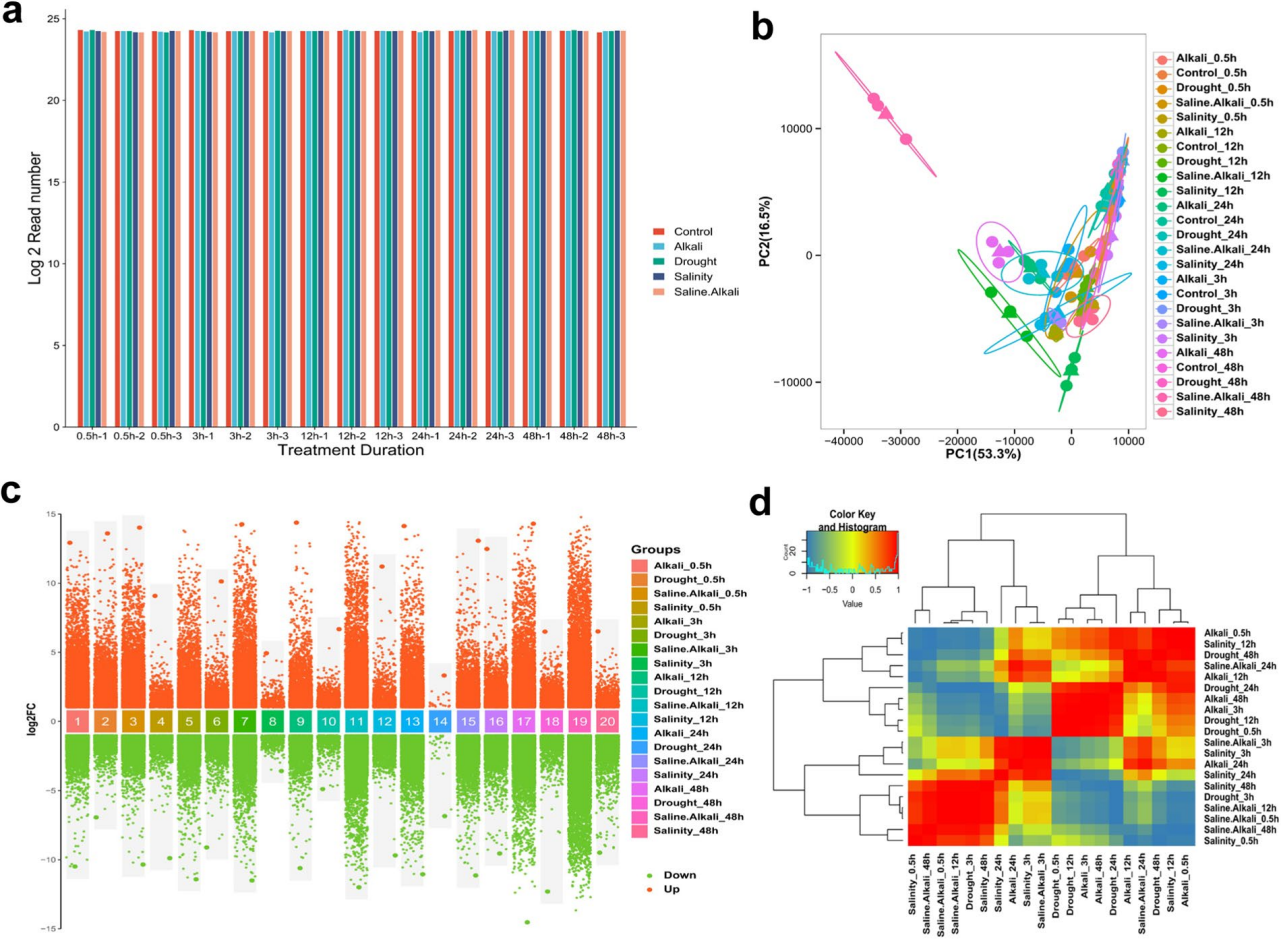
Transcriptome data assessment. (**a**) Total number of pair-end reads in clean data; (**b**) Principal component analysis of all the stresses (drought, salinity, alkali, and saline-alkali stress) in each time period; (**c**) Log2FC-based number of DEGs plot. The number of up and down-regulated genes are shown in orange and green color, respectively; (**d**) Analysis of correlation of the clean reads in each stress at each time point. (Preferably placed before Transcriptome data analysis).

**Fig. 3:**
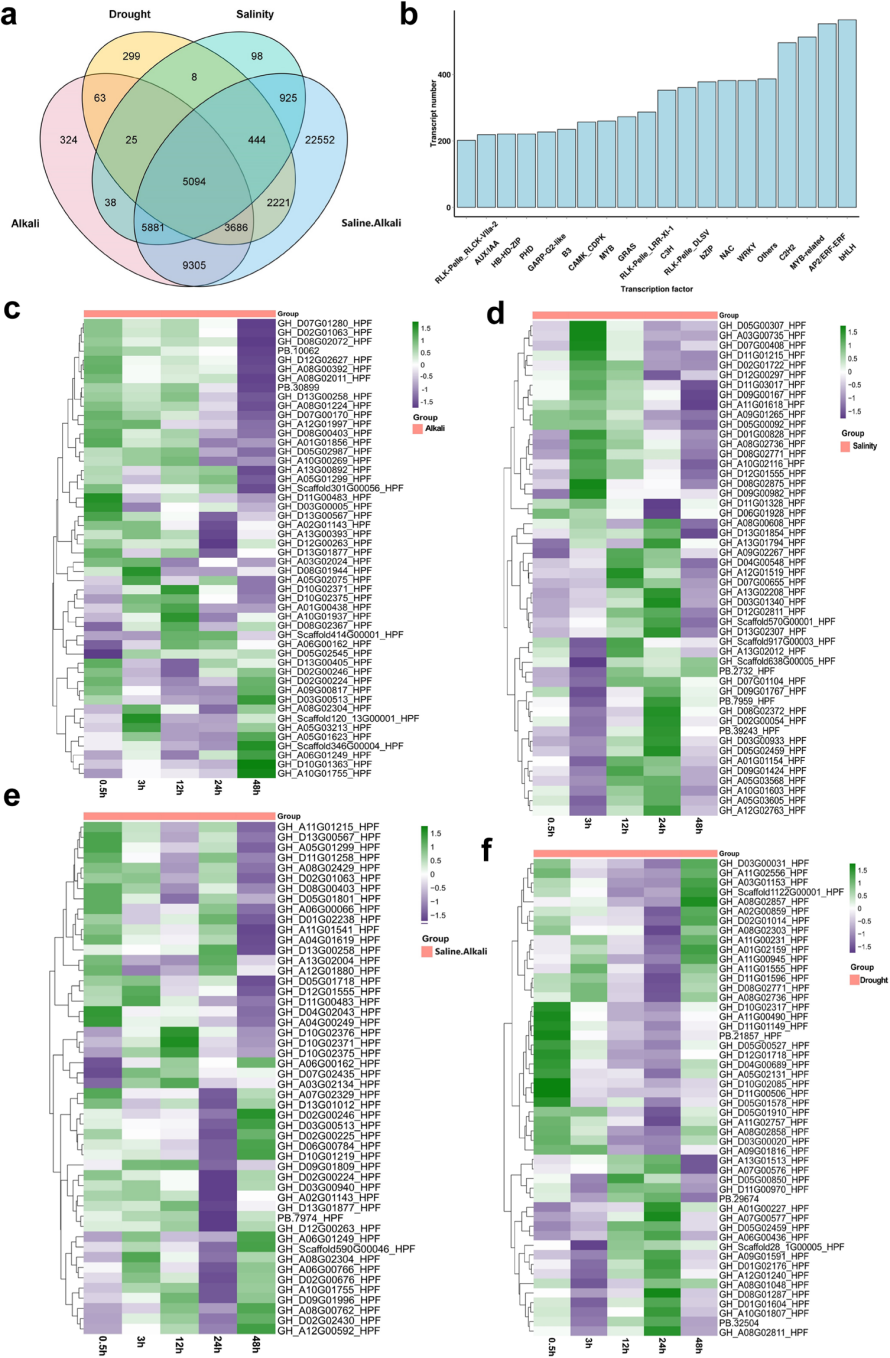
Differential gene expression (DEGs) analysis in *G. purpurascnes* (**a**) A Venn diagram for the total number of shared DEGs between drought, salinity, alkali, and saline-alkali stresses; (**b**) Transcription factors and their associated numbers of the transcript; Expression patterns of top 45 DEGs in alkali stress (**c**); salinity stress (**d**); saline-alkali stress; (**e**) drought stress (**f**). The green color indicates upregulation, and the purple color reflects down-regulation while the orange colour indicates groups. (Preferably placed before Annotation).

The goal was to measure the expression of genes under different environmental stresses in cotton. This was accomplished by converting the number of reads obtained from sequencing into read counts for each cotton gene. To compare the distributions of normalized read counts across all samples, a bar graph has been created and presented in Fig. 2a. The graph provides a clear representation of the variations in normalized read counts among the samples. The results of the principal component analysis showed that the top two main components were responsible for most of the variations observed in the data. Furthermore, the samples from each treatment group exhibited comparable patterns and were found to be clustered together, as illustrated in Fig. 2b. The high values of PC1 and PC2 serve as an affirmation of the data’s quality and indicate that a significant portion of the observed variation is attributed to the diverse treatments being studied. The co-occurrence of multiple treatments demonstrated a strong positive correlation, indicating that an increase in one treatment was often accompanied by an increase in another treatment. We conducted a thorough exploration of the gene expression patterns by carrying out an analysis of the DEGs that are linked with each abiotic stress. Furthermore, we compared the findings of these analyses with those of the control to gain a comprehensive understanding of the underlying mechanisms. The graphical representation in Fig. 2c displays the logarithmic fold change of the transformed data along the vertical axis. The upregulated genes are marked in orange, while the downregulated genes are highlighted in green. It is worth noting that the application of saline alkali treatment for 48 hours resulted in the most significant changes in gene expression levels, with the highest number of differentially expressed genes observed both up and down. While the lowest number of up and down DEGs were represented by drought treatment at 24 h. An examination was conducted to determine the association between clean reads, and it was discovered that there was a significant correlation for each stress level at a later stage (Fig. 2d). Among all the treatments, saline alkali treatments showed the strongest positive correlation with the other treatments at different time points. The Venn diagram representing DEGs in various stresses established that the highest number of DEGs were detected in the saline-alkali stress condition. (Fig. 3a). A total of 5094 DEGs were identified as common across all the treatments. Saline-alkali treatment induced the unique highest number of DEGs (22552) compared to other stress treatments such as alkali (324), drought (299), and salinity (98). Moreover, the present study has brought to light that the presence of a higher number of bHLH, AP2/ERF, and MYb-related transcription factors may indicate their significant contribution in enhancing the plant’s ability to withstand abiotic stress (Fig. 3b). The heatmap visually represents the gene expression patterns for the top forty-five most significant DEGs. This helps identify the genes most strongly associated with a particular stress namely alkali, salinity, saline-alkali and drought (Fig. 3c–f). We employed a log FC greater than 2 with a false discovery rate threshold (FDR < 0.001) and a Pvalue < 0.005 to identify the DEGs. Our analysis revealed 205,403 DEGs that were either unique or shared between the different stress treatments.

### Annotation

**Fig. 4:**
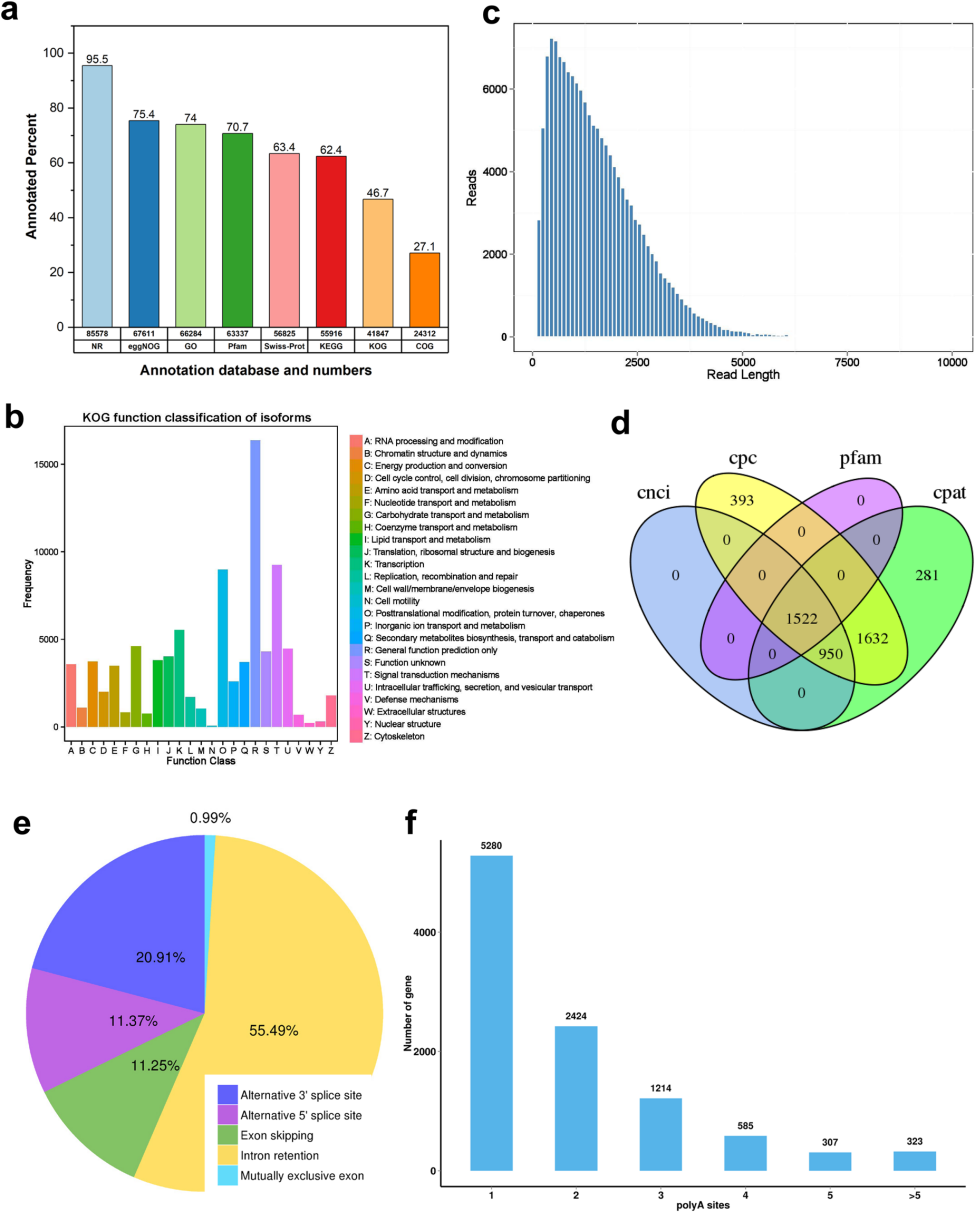
Prediction of functions of ISO-seq isoform in *G. purpurascens* (**a**) Annotation databases and their corresponding percentages; (**b**) KOG function classification isoforms with their frequency; (**c**) cDNA read length with their number of reads; (**d**) The Venn diagram of predicted long non-coding RNA (lncRNA) of iso-seq isoforms; (**e**) Alternative splicing events of iso-seq isoforms; (**f**) Alternative polyadenylation sites of iso-seq isoforms. (Preferably placed before Usage Notes).

**Fig. 5:**
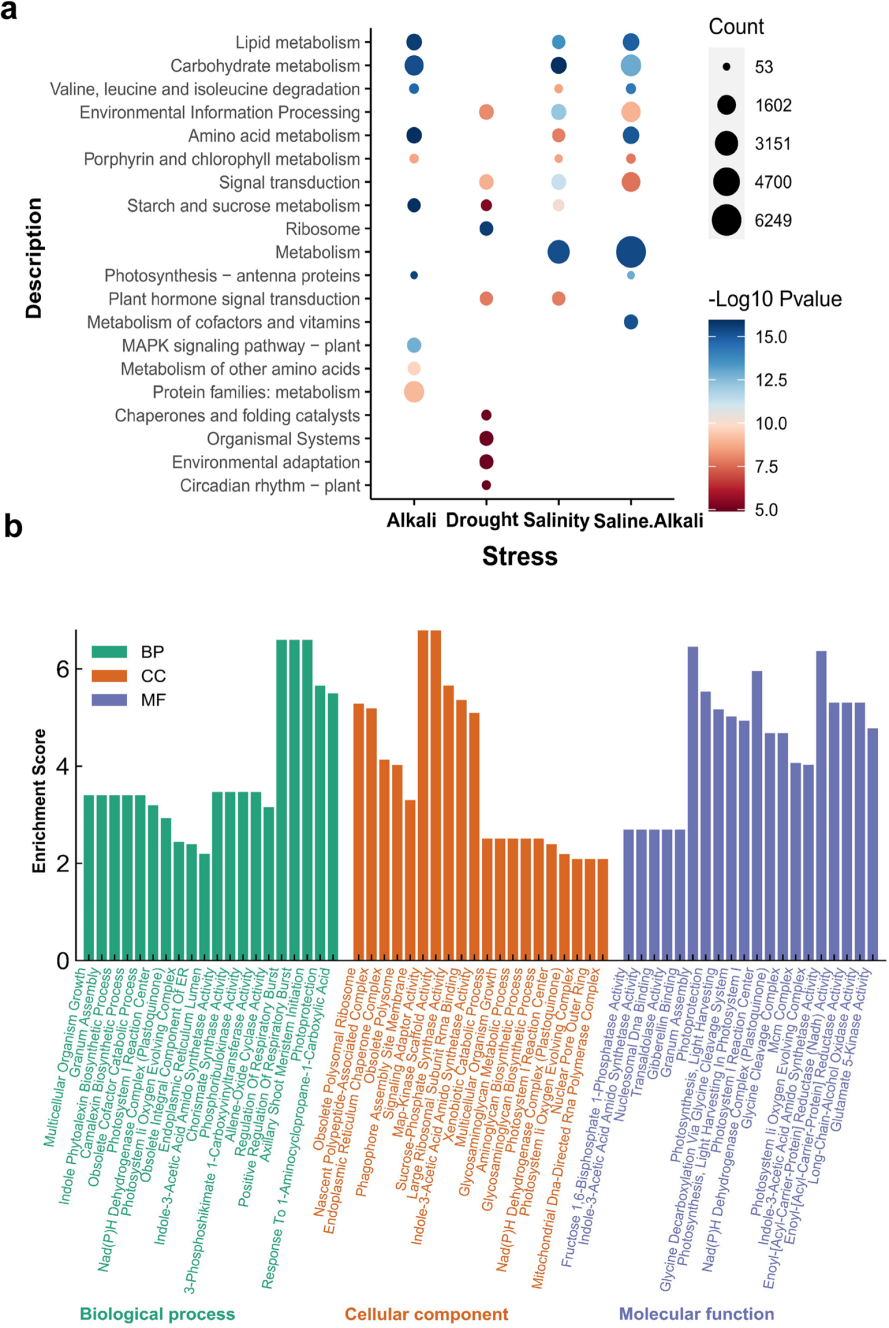
Enrichment analysis of DEGs of alkali, drought, salinity and saline-alkali stress stresses. (**a**) KEGG analysis of differentially expressed genes (DEGs) in terms of Metabolism, Organismal Systems, Genetic Information Processing, and Environmental Information Processing. The term Count displays the count of genes that are linked with every KEGG term. The intensity of blue color indicates the highest, while that of red color shows the lowest negative log_10_ *P value*; (**b**) Gene ontology identifying cellular components, molecular functions and biological processes. The left side of the display shows the percentage of genes associated with each ontology term, while the right side demonstrates the percentage of genes in the dataset that are associated with that term. (Preferably placed before Usage Notes).

In the study of *G. purpurascens*, full-length transcripts have been annotated using multiple reference databases to enable in-depth analysis. Out of the total transcripts analyzed, 85,578 (95.5%), 67,611 (75.4%), and 66,284 (74.0%) transcripts show significant similarity with the sequences available in NCBI non-redundant protein sequences (NR), eggNOG, and GO database, respectively. Moreover, Pfam and Swiss-Port databases annotated 70.7% (63,337) and 63.4% (56,825) transcripts, respectively (Fig. 4a). Whereas the process of function classification was performed on ISO isoforms by mapping them to the KOG database (Fig. 4b). The most frequently observed functional category among isoforms was the general function prediction class, followed by signal transduction mechanism and post-translational modifications. The read length distribution of iso-seq isoforms and the number of reads is depicted in Fig. 4c. Non-coding RNAs (lncRNAs) are crucial plant growth and development regulators. They are involved in various biological processes, epigenetic modifications, and signal transduction pathways. LncRNAs have emerged as essential players in plant biology and are being extensively studied to unravel their complex roles and functions. In this study, candidate lncRNAs were identified using PFAM, CPC, CNCI, and PLEK databases. These databases were employed to predict 1522 lncRNAs^45^ based on their coding potential, sequence features, and homology to known protein domains (Fig. 4d). The PacBio dataset analysis revealed a total of 20593 alternative splicing (AS) events and further classified into five types. AS validated intron retention (55.49%) is the most prevalent event, followed by alternative 3′ splice site (20.91%), alternative 5′ splice site (11.37%), exon skipping (11.25%) and mutually exclusive exon (0.99%) (Fig. 4e). The Iso-Seq analysis detected a total of 10133 genes which contain at least one poly(A) site. Out of these, 52.11% (5280) genes contained only one poly(A) site, while 23.92% (2424) genes had two poly(A) sites (Fig. 4f). Whereas 2429 genes were found to have three or more poly(A) sites. This indicates that these particular genes may have a complex regulation mechanism, which involves multiple polyadenylation events. We performed KEGG enrichment analysis to validate plant responses to different abiotic stress treatments. Our study revealed stress-responsive KEGG enrichments^41^ and identified conserved and unique KEGG terms for each treatment (Fig. 5a). The gene expression patterns and GO enrichments observed in our data indicate their potential usefulness in comparing gene expression changes under different abiotic stress conditions (Fig. 5b). The gene expression patterns and GO and KEGG enrichments observed in our data provide valuable insights into how different abiotic stresses interact with each other over time, allowing us to understand the evolutionary mechanisms underlying the adaptation of cotton to harsh environments.

## Usage Notes

The Iso-seq data is a powerful tool that enables researchers to accurately measure the sequence and assembly of full-length transcripts, as well as identify alternative splicing events, alternative polyadenylation sites, simple sequence repeats (SSR), novel coding isoforms, and lncRNA sequences. This technology has been applied to study five different treatments in *G. purpurascens*, providing valuable insights into the genetic mechanisms behind this species’ response to various environmental stimuli. In addition, paired-end RNA-seq data can identify and sequence RNA molecules and also serve a valuable function in the quantification of gene expression and the evaluation of alternative isoform usage. This capability makes it a convincing tool for analyzing the complexity of gene expression and distinguishing the specific processes that underlie the transcriptional regulation of genes.

During the study, *G. purpurascens* was subjected to various abiotic stress conditions such as drought, salinity, saline-alkali, and alkali stress to evaluate its tolerance to such stress factors. The aim was to investigate the plant’s ability to withstand harsh environmental conditions. The available data set comprises both long-read and short-read sequencing data, providing a comprehensive and diverse sequencing resource. This rich dataset offers ample opportunities for enumerating gene or transcript expression, exploring novel transcripts, assessment of alternative splicing, refining the annotation of the cotton genome, and uncovering the genetic mechanisms responsible for abiotic stress tolerance in cotton. With its potential for extensive analysis, this dataset is a valuable resource for researchers in the field of cotton genomics.

### Supplementary information

Supplementary Table S1

## Data Availability

The study utilized publicly available software with clear methodological descriptions of their parameters. In cases where no specific parameters were provided, we opted to use the default parameters as suggested by the software developer.
