# Supplementary material for: Transcriptome dynamics of Gossypium purpurascens in response to abiotic stresses by Iso-seq and RNA-seq data
Source: Sci Data. 2024 May 9;11:477. doi: 10.1038/s41597-024-03334-9 (PMC11081948; doi:10.1038/s41597-024-03334-9)
Supplement: Supplementary file 1 — Supplementary Table S1 [file 41597_2024_3334_MOESM1_ESM.docx]

**Transcriptome dynamics of *Gossypium purpurascens* in response to abiotic stresses by Iso-seq and RNA-seq data**
Abdul Rehman^1^, Chunyan Tian^1^, Shoupu He^1,2^, Hongge Li^1,2^, Shuai Lu^4^, Xiongming Du^1,2,3^*, Zhen Peng^1,2,3^*
1. Zhengzhou Research Base, State Key Laboratory of Cotton Bio-breeding and Integrated Utilization, School of Agricultural Sciences, Zhengzhou University, Zhengzhou, 450001, China.
2. State Key Laboratory of Cotton Bio-breeding and Integrated Utilization, Institute of Cotton Research, Chinese Academy of Agricultural Sciences (ICR, CAAS), Anyang, Henan 455000, China.
3. National Nanfan Research Institute (Sanya), Chinese Academy of Agricultural Sciences, Sanya, Hainan 572024, China.
4. National Supercomputing Center in Zhengzhou, Zhengzhou University, Zhengzhou, 450001, China.
*Correspondence:
Dr. XM. Du: E-mail: duxiongminglab@caas.cn;
Dr. Zhen Peng: E-mail: pengzhen01@caas.cn; Tel: +86-0372-2562252

**Supplementary Table S1: Summary of NGS data alignment against the reference genome.**

| **Run title** | **Description** | **Clean Reads** | **Mapped reads (%)** | **Uniquely mapped reads (%)** | **GC Content (%)** | **Q30 (%)** | **Biosample accession** |
| --- | --- | --- | --- | --- | --- | --- | --- |
| K411-AS-0.5h-1 | 0.5h, Alkali_1 | 39,661,410 | 96.64 | 90.22 | 43.7 | 93.7 | CRR1014777 |
| K411-AS-0.5h-2 | 0.5h, Alkali_2 | 40,448,262 | 96.71 | 90.23 | 43.77 | 93.68 | CRR1014778 |
| K411-AS-0.5h-3 | 0.5h, Alkali_3 | 39,170,486 | 96.51 | 90.03 | 43.65 | 93.7 | CRR1014779 |
| K411-CK-0.5h-1 | 0.5h, Control_1 | 42,313,992 | 96.77 | 90.39 | 44.04 | 93.69 | CRR1014771 |
| K411-CK-0.5h-2 | 0.5h, Control_3 | 40,458,794 | 96.31 | 89.94 | 44.13 | 93.62 | CRR1014772 |
| K411-CK-0.5h-3 | 0.5h, Control_2 | 40,260,738 | 96.77 | 90.25 | 44.11 | 94.19 | CRR1014773 |
| K411-DS-0.5h-1 | 0.5h, Drought_1 | 42,430,592 | 96.91 | 90.52 | 44.16 | 94.39 | CRR1014783 |
| K411-DS-0.5h-2 | 0.5h, Drought_2 | 40,460,218 | 96.73 | 90.37 | 44.15 | 93.41 | CRR1014784 |
| K411-DS-0.5h-3 | 0.5h, Drought_3 | 38,102,628 | 97.06 | 90.58 | 44.15 | 94.3 | CRR1014785 |
| K411-SAS-0.5h-1 | 0.5h, Saline-Alkali_1 | 38,979,498 | 96.66 | 90.47 | 43.76 | 93.95 | CRR1014780 |
| K411-SAS-0.5h-2 | 0.5h, Saline-Alkali_2 | 38,370,774 | 96.3 | 90.05 | 43.79 | 93.72 | CRR1014781 |
| K411-SAS-0.5h-3 | 0.5h, Saline-Alkali_3 | 40,424,048 | 95.27 | 89.09 | 43.98 | 91.27 | CRR1014782 |
| K411-SS-0.5h-1 | 0.5h, Salinity_1 | 40,555,576 | 96.77 | 90.5 | 43.75 | 93.75 | CRR1014774 |
| K411-SS-0.5h-2 | 0.5h, Salinity_2 | 38,497,566 | 96.63 | 90.25 | 43.8 | 93.64 | CRR1014775 |
| K411-SS-0.5h-3 | 0.5h, Salinity_3 | 40,814,488 | 96.64 | 90.31 | 43.64 | 93.54 | CRR1014776 |
| K411-AS-3h-1 | 3h, Alkali_1 | 40,625,286 | 96.39 | 90.11 | 43.77 | 93.66 | CRR1014792 |
| K411-AS-3h-2 | 3h, Alkali_2 | 40,092,828 | 96.39 | 90.19 | 43.76 | 93.6 | CRR1014793 |
| K411-AS-3h-3 | 3h, Alkali_3 | 38,335,168 | 96.56 | 90.22 | 43.76 | 93.63 | CRR1014794 |
| K411-CK-3h-1 | 3h, Control_1 | 42,060,570 | 97.34 | 90.58 | 44.21 | 92.95 | CRR1014786 |
| K411-CK-3h-2 | 3h, Control_3 | 40,086,702 | 96.63 | 90 | 44.19 | 93.44 | CRR1014787 |
| K411-CK-3h-3 | 3h, Control_2 | 40,473,120 | 95.85 | 89.31 | 44.04 | 93.56 | CRR1014788 |
| K411-DS-3h-1 | 3h, Drought_1 | 40,448,406 | 96.68 | 90.13 | 44.09 | 93.87 | CRR1014798 |
| K411-DS-3h-2 | 3h, Drought_2 | 40,085,324 | 96.23 | 89.63 | 44.23 | 93.42 | CRR1014799 |
| K411-DS-3h-3 | 3h, Drought_3 | 41,148,304 | 96.86 | 90.35 | 44.2 | 93.84 | CRR1014800 |
| K411-SAS-3h-1 | 3h, Saline-Alkali_1 | 38,135,788 | 95.96 | 89.78 | 43.74 | 93.85 | CRR1014795 |
| K411-SAS-3h-2 | 3h, Saline-Alkali_2 | 40,427,048 | 96.6 | 90.41 | 43.62 | 93.57 | CRR1014796 |
| K411-SAS-3h-3 | 3h, Saline-Alkali_3 | 40,328,508 | 96.45 | 90.37 | 43.55 | 93.32 | CRR1014797 |
| K411-SS-3h-1 | 3h, Salinity_1 | 38,994,510 | 96.47 | 90.03 | 43.82 | 93.32 | CRR1014789 |
| K411-SS-3h-2 | 3h, Salinity_2 | 40,207,576 | 96.35 | 89.79 | 43.98 | 93.34 | CRR1014790 |
| K411-SS-3h-3 | 3h, Salinity_3 | 40,169,720 | 96.55 | 90.01 | 43.98 | 93.57 | CRR1014791 |
| K411-AS-12h-1 | 12h, Alkali_1 | 40,394,910 | 96.56 | 90.35 | 43.15 | 93.06 | CRR1014807 |
| K411-AS-12h-2 | 12h, Alkali_2 | 42,333,736 | 96.28 | 90.12 | 43.19 | 93.51 | CRR1014808 |
| K411-AS-12h-3 | 12h, Alkali_3 | 40,623,304 | 96.38 | 90.17 | 43.45 | 93.29 | CRR1014809 |
| K411-CK-12h-1 | 12h, Control_1 | 40,407,002 | 96.72 | 90.1 | 43.48 | 94.09 | CRR1014801 |
| K411-CK-12h-2 | 12h, Control_3 | 40,668,762 | 96.84 | 90.39 | 43.41 | 93.87 | CRR1014802 |
| K411-CK-12h-3 | 12h, Control_2 | 40,612,630 | 96.45 | 89.96 | 43.53 | 92.99 | CRR1014803 |
| K411-DS-12h-1 | 12h, Drought_1 | 40,460,860 | 96.83 | 90.46 | 43.47 | 93.95 | CRR1014813 |
| K411-DS-12h-2 | 12h, Drought_2 | 40,357,406 | 95.77 | 89.42 | 43.54 | 93.38 | CRR1014814 |
| K411-DS-12h-3 | 12h, Drought_3 | 40,190,086 | 96.72 | 90.33 | 43.54 | 93.45 | CRR1014815 |
| K411-SAS-12h-1 | 12h, Saline-Alkali_1 | 40,389,024 | 96.43 | 90.43 | 43.65 | 93.29 | CRR1014810 |
| K411-SAS-12h-2 | 12h, Saline-Alkali_2 | 40,121,068 | 95.97 | 90.04 | 43.63 | 92.8 | CRR1014811 |
| K411-SAS-12h-3 | 12h, Saline-Alkali_3 | 41,052,220 | 96.15 | 90.15 | 43.53 | 93.03 | CRR1014812 |
| K411-SS-12h-1 | 12h, Salinity_1 | 40,606,592 | 96.94 | 90.51 | 43.47 | 93.82 | CRR1014804 |
| K411-SS-12h-2 | 12h, Salinity_2 | 40,861,430 | 96.39 | 90.14 | 43.32 | 93.23 | CRR1014805 |
| K411-SS-12h-3 | 12h, Salinity_3 | 40,432,508 | 96.71 | 90.49 | 43.45 | 93.76 | CRR1014806 |
| K411-AS-24h-1 | 24h, Alkali_1 | 38,600,196 | 96.28 | 90.29 | 43.66 | 93.34 | CRR1014822 |
| K411-AS-24h-2 | 24h, Alkali_2 | 41,456,842 | 96.81 | 90.8 | 43.74 | 94.06 | CRR1014823 |
| K411-AS-24h-3 | 24h, Alkali_3 | 40,523,198 | 97.16 | 91.07 | 43.74 | 94.45 | CRR1014824 |
| K411-CK-24h-1 | 24h, Control_1 | 40,615,680 | 96.77 | 90.4 | 43.8 | 93.55 | CRR1014816 |
| K411-CK-24h-2 | 24h, Control_3 | 40,381,188 | 95.92 | 89.63 | 44.19 | 91.81 | CRR1014817 |
| K411-CK-24h-3 | 24h, Control_2 | 40,342,222 | 96.58 | 90.29 | 43.94 | 93.27 | CRR1014818 |
| K411-DS-24h-1 | 24h, Drought_1 | 41,106,344 | 96.88 | 90.59 | 44.06 | 94.06 | CRR1014828 |
| K411-DS-24h-2 | 24h, Drought_2 | 41,447,572 | 96.78 | 90.33 | 43.79 | 93.67 | CRR1014829 |
| K411-DS-24h-3 | 24h, Drought_3 | 39,581,768 | 97.17 | 90.78 | 43.86 | 94.29 | CRR1014830 |
| K411-SAS-24h-1 | 24h, Saline-Alkali_1 | 41,524,174 | 96.39 | 90.3 | 43.81 | 93.37 | CRR1014825 |
| K411-SAS-24h-2 | 24h, Saline-Alkali_2 | 42,290,442 | 96.87 | 90.86 | 43.58 | 93.78 | CRR1014826 |
| K411-SAS-24h-3 | 24h, Saline-Alkali_3 | 41,770,036 | 96.89 | 90.9 | 43.62 | 93.21 | CRR1014827 |
| K411-SS-24h-1 | 24h, Salinity_1 | 40,144,636 | 96.59 | 90.2 | 43.59 | 93.52 | CRR1014819 |
| K411-SS-24h-2 | 24h, Salinity_2 | 40,995,072 | 96.74 | 90.69 | 43.2 | 93.27 | CRR1014820 |
| K411-SS-24h-3 | 24h, Salinity_3 | 41,483,994 | 94.9 | 88.95 | 43.55 | 90.02 | CRR1014821 |
| K411-AS-48h-1 | 48h, Alkali_1 | 40,754,794 | 96.6 | 90.4 | 43.51 | 92.35 | CRR1014837 |
| K411-AS-48h-2 | 48h, Alkali_2 | 40,700,882 | 97.39 | 91.38 | 43.53 | 93.99 | CRR1014838 |
| K411-AS-48h-3 | 48h, Alkali_3 | 40,412,772 | 97.07 | 90.96 | 43.61 | 94.09 | CRR1014839 |
| K411-CK-48h-1 | 48h, Control_1 | 40,668,736 | 96.66 | 90.44 | 43.89 | 94.2 | CRR1014831 |
| K411-CK-48h-2 | 48h, Control_3 | 40,901,696 | 96.49 | 90.22 | 44.04 | 93.85 | CRR1014832 |
| K411-CK-48h-3 | 48h, Control_2 | 38,153,466 | 97.25 | 91.07 | 43.91 | 94.11 | CRR1014833 |
| K411-DS-48h-1 | 48h, Drought_1 | 40,685,208 | 96.71 | 90.49 | 44.11 | 93.58 | CRR1014843 |
| K411-DS-48h-2 | 48h, Drought_2 | 42,214,240 | 97.2 | 90.49 | 44.13 | 92.76 | CRR1014844 |
| K411-DS-48h-3 | 48h, Drought_3 | 40,454,300 | 96.9 | 90.92 | 44.19 | 93.43 | CRR1014845 |
| K411-SAS-48h-1 | 48h, Saline-Alkali_1 | 40,833,922 | 97.21 | 91.26 | 43.92 | 94.02 | CRR1014840 |
| K411-SAS-48h-2 | 48h, Saline-Alkali_2 | 40,359,436 | 96.32 | 90.38 | 44 | 92.54 | CRR1014841 |
| K411-SAS-48h-3 | 48h, Saline-Alkali_3 | 41,125,484 | 97.43 | 91.21 | 43.91 | 93.37 | CRR1014842 |
| K411-SS-48h-1 | 48h, Salinity_1 | 40,682,994 | 97.16 | 90.97 | 43.82 | 93.72 | CRR1014834 |
| K411-SS-48h-2 | 48h, Salinity_2 | 40,629,230 | 96.93 | 90.8 | 43.67 | 93.08 | CRR1014835 |
| K411-SS-48h-3 | 48h, Salinity_3 | 41,308,344 | 95.98 | 89.78 | 43.73 | 93.79 | CRR1014836 |
